# Out-of-home activity participation associated with body mass index and muscle mass in community-dwelling older adults

**DOI:** 10.1186/s12889-025-24813-7

**Published:** 2025-12-12

**Authors:** Lumi Kinjo, Yee Sien Ng, Sapphire Lin, Helen Hoenig

**Affiliations:** 1 Duke-NUS Medical School, 8 College Road, Singapore, 169857 Singapore; 2 Singapore General Hospital, Singapore, Singapore; 3 Centre for Population Health Research and Implementation, Singapore Health Services, Singapore, Singapore; 4 The Institute for Digital Medicine, Yong Loo Lin School of Medicine, National University of Singapore, Singapore, Singapore; 5 The N.1 Institute for Health, National University of Singapore, Singapore, Singapore; 6 Physical Medicine and Rehabilitation Service, Durham Veterans Affairs Medical Center, Durham, NC USA; 7 Duke Aging Center, Duke University School of Medicine, Durham, NC USA

**Keywords:** Older Adults, Community Activities, BMI, Muscle Mass

## Abstract

**Background:**

Extremes of body mass index (BMI) and low Muscle Mass are associated with increased morbidity and mortality in older adults. Modifiable factors may include the frequency of participation in key out-of-home activities.

**Methods:**

Using a cross-sectional study design, we examined the relationship between frequencies of out-of-home activities—specifically dining, grocery shopping, exercise, and recreation—and BMI as well as Muscle Mass among community-dwelling adults aged 50 years and older in Singapore (*n* = 1,118). Frequencies of out-of-home activities over 14 days were measured using Global Positioning System (GPS)-based travel data. Participants’ sociodemographic, health, and neighborhood environmental information was collected through a questionnaire, physical performance tests, and publicly available databases. Multivariable regression was performed to examine the association between activity frequency and BMI as well as Muscle Mass while controlling for known predictors of the outcome variables.

**Results:**

We found a positive and significant association between out-of-home dining frequency and BMI even after controlling for exercise frequency and self-reported physical activity level. Out-of-home exercise and recreation frequencies were also associated with Muscle Mass. Importantly, increased participation in out-of-home recreational activities was significantly associated with higher Muscle Mass, independent of out-of-home exercise frequency and self-reported physical activity level. Other findings included association between low gait speed and higher BMI. Lower perceived income adequacy was also associated with higher BMI, after controlling for income surrogates such as housing type, education, and employment status. Living in a neighborhood with hills and/or barriers to walking from place to place was associated with greater Muscle Mass. However, most environmental factors including distance to nearest dining cluster and supermarket were not associated with BMI or Muscle Mass.

**Conclusions:**

Key modifiable life activities associated with clinically significant differences for BMI include out-of-home dining frequency, whereas for Muscle Mass, they include out-of-home recreation and exercise frequencies.

**Trial registration:**

Not applicable.

**Supplementary Information:**

The online version contains supplementary material available at 10.1186/s12889-025-24813-7.

## Introduction

Aging is associated with decreased fat-free mass and increased adipose tissue [1]. Extremes of high and low body mass index (BMI) are linked to increased morbidity [2] and mortality [3]. Low Muscle Mass is associated with adverse health outcomes, and when sarcopenia coexists with obesity, there is greater risk of morbidity and mortality than with either condition alone [4].

While many factors contribute to human body composition, diet and physical activity are the most pertinent factors influencing both BMI [5] and Muscle Mass [6]. However, isolated dietary and exercise regimens have low long-term adherence [7]. Understanding how daily life activities related to diet and physical activity impact BMI and Muscle Mass could have substantial clinical implications, as it may be easier to modify.

Regarding the impact of food-related activities on BMI and Muscle Mass, existing studies are mainly focused on the association between body weight and dining out frequency. The results are inconclusive with conflicting outcomes [8]. Most of these studies did not use objective measures of assessing out-of-home dining frequency, nor did they account for other types of daily activity (e.g., grocery shopping, exercise, recreation) or environmental factors. Others have studied the association between BMI and proximity or density of fast-food restaurants [9, 10] and supermarkets [9] and found significant associations. However, these studies did not consider actual dining and grocery shopping frequency. Therefore, the relationship between out-of-home dining frequency and BMI should be studied further, with consideration of environmental covariates and other life activities. Studies on the relationship between BMI and grocery shopping frequency are lacking to date, as are studies on the relationship between Muscle Mass and dining out or grocery shopping frequency.

Regarding the importance of physical activity on BMI and Muscle Mass, the association between exercise with lower BMI [11] and improved Muscle Mass [12] are well established. Growing evidence also suggest the importance of non-exercise lifestyle activities on BMI and Muscle Mass. For example, leisure time sitting is associated with higher obesity risk independent of physical activity [13]. While there are many studies on the effect of leisure-time physical activity and BMI [14, 15], leisure-time physical activity is most often defined as sports, exercise, walking, jogging, or cycling, which could be broadly categorized under “exercising” and does not account for other activities that people engage in for pleasure and recreation. In fact, there are only few studies on the relationship between non-exercise leisure activities and BMI or Muscle Mass. For instance, one study found that gardening was negatively associated with body fat and positively associated with appendicular skeletal muscle mass in community-dwelling older men [16]. Another study found a negative correlation between the frequency of attending entertainment and BMI among community-dwelling older adults [17]. In both studies, however, participation was measured based on questionnaire rather than objective activity data. Moreover, participation in other potentially influential out-of-home life activities such as dining and grocery shopping were not considered. There is a need to further understand the impact of non-exercise recreational activity on body composition in community-dwelling older adults using objective measures of activity participation.

In this study, we investigated the association between frequencies of key out-of-home activities—specifically dining, grocery shopping, exercise, and recreation—and BMI as well as Muscle Mass among community-dwelling older adults. The activities were selected due to their relevance to diet and physical activity levels, which are the most pertinent factors that affect BMI and Muscle Mass. We employed GPS-based travel data to objectively quantify activity frequencies, while accounting for sociodemographic, health, and environmental factors.

## Methods

### Study population

This study is based on data collected in the Elderly Life Activity-Space (EASE) study, a large cross-sectional study of community-dwelling older adults in Singapore. Individuals aged 50 and older who live within the geographical boundary corresponding to the areas served by Singapore Health Services (SingHealth) were recruited (*n* = 1,131). Participants were recruited to reflect the nation’s housing type distribution, retirement age, and prevalence of frail status in the community. All participants who completed the study were provided with grocery vouchers equivalent to SGD 150.

### Data collection

In-person data collection was conducted at seven community sites between April 2022 through August 2023. Data was collected by three means: a questionnaire, physical performance tests, and GPS-based travel data over 14-days. Informed consent was obtained from all participants. The SingHealth Centralized Institutional Review Board reviewed and approved the ethics of this study [CIRB Reference Number: 2021/2566].

### Variables

#### Outcome variables

The two outcome variables in this study were BMI and Muscle Mass. BMI was calculated by dividing the weight by height squared. Weight and height were measured with a digital scale and a stadiometer. Muscle Mass was measured using a multi-frequency segmental bioelectrical impedance analysis (BIA), a widely accepted method for measuring Muscle Mass by Asian Consensus [18], and was calculated by dividing the sum of muscle mass of the four limbs by height squared. Participants with metal implant(s) were excluded from BIA measurements.

#### Main independent variables

##### Out-of-home activity stop counts

The main independent variable of our study was how often each participant engaged in a particular out-of-home activity over the course of 14-day data collection period. This information was collected using a GPS-based mobility sensing application downloaded on each participant’s smartphone. Each participant kept a travel diary using this application in which they were asked to record *where* they went (‘stops’) and *why* they visited the stops (‘activities’). The purpose of each stop was presented as a list of activities in which the participants could select from. The activities of our interest were (1) eating out at public places (‘dining’), (2) shopping for daily necessities at a store (‘grocery shopping’), (3) exercising, working out, and playing sports (‘exercise’), and (4) attending activities for entertainment or fun (‘recreation’). We derived the out-of-home activity frequency by summing the number of ‘stops’ for a given activity over the 14-days. For example, the out-of-home dining frequency is the sum of number of ‘stops’ in which the recorded activity was ‘dining’ (‘Total Stop Count for Dining’).

The application system employed in our study has been used in multiple cities across the world [19]. It allows for the collection of high-resolution travel data for each participant and mitigates the shortcomings of traditional recall-based travel surveys such as under-reporting of trips. To ensure the accuracy of travel data, all participants were guided through the initial installation and usage of the smartphone application in-person. They were also provided with written instructions. The application had built-in features to encourage user compliance and minimize data entry errors, for example, having automated reminders and not allowing overlapping timestamps or missing data fields. Smartphone-based travel logs were reviewed, and participants were followed up if they seemed to be having any difficulty. The 14-day duration was chosen to sufficiently capture participants’ activity patterns [20].

If the participant was unable or declined to use the smartphone application, travel information was recorded on paper. Those using paper-based travel logs were also contacted every 2–3 days to be reminded to record their travels.

#### Covariates

To determine which covariates should be included in the analyses, all variables in the database were reviewed and potential predictors for BMI and Muscle Mass were identified a priori, guided by existing literature and clinical judgement. The predictors of BMI or Muscle Mass included in our analyses included sociodemographic, environmental, and health-related variables. The same set of predictor variables were used for BMI and Muscle Mass with two exceptions. Muscle Mass was used as a predictor only for BMI and MNA score was used as a predictor only for Muscle Mass since BMI is a component of MNA.

##### Sociodemographic variables

Sociodemographic covariates in our model included age [21–24], gender [21, 24], race [21, 24], education [22–24], employment status [22], perceived income adequacy, housing type, marital status [10, 24, 25], and number of household members [23]. Social network [26, 27] was assessed using the abbreviated 6-item Lubben Social Network Scale (LSNS-6) [28].

##### Environmental variables

Environmental attributes associated with BMI and Muscle Mass include distance from home to supermarket/grocery store [9, 10], bus stop density around home [29], distance from home to senior activity center [29], availability of parks and spaces for physical activity around home [30], street network in the neighborhood [31], residential density [32], and neighborhood walkability [33]. We included covariates similar or equivalent to these variables in our model. Most environmental variables were derived based on open-source databases such as OneMap, Data.gov.sg, and LTA DataMall. Distance from home to the nearest dining options was calculated by clustering the dining options using the Density-Based Spatial Clustering of Applications with Noise (DBSCAN) [34] algorithm, with clustered points separated by a maximum of 25 m. Additional variables included distance from participants’ homes to the nearest supermarket, wet market, senior activity center, park, and fitness corner. Any green space ≥ 1,000m^2^ was considered as a park. Density variables were also included, which consisted of number of bus stops within a 400 m radius, percentage of park areas within a 1 km radius, total road network length within a 400 m radius, and number of dwelling units within a 1 km radius, all measured from participants’ homes. Our model also included a subscale from the Neighborhood Environment Walkability Scale - Abbreviated (NEWS-A) questionnaire [35]. It measured perceived barriers to a walkable neighborhood based on the mean of responses to the following two questions: ‘The streets in my neighborhood are hilly, making my neighborhood difficult to walk in’ and ‘There are major barriers to walking in my local area that make it hard to get from place to place (e.g., freeways, railway lines, rivers)’.

##### Health variables

Health variables were derived from physical performance tests and a questionnaire. We included predictors for BMI and Muscle Mass in our models, including gait speed [36], loneliness [25], depressive symptoms [25, 37], Mini Nutritional Assessment (MNA) score [38], physical activity level [5, 6], and Muscle Mass [39]. Gait speed was calculated by measuring the time participants took to walk a 10 m course at their usual walking speeds, with 2 m placed before and after the course for acceleration and deceleration. Participants underwent two trials, and the faster gait speed was used for analysis. The cut-off for low gait speed was < 1.0 m/s [18]. Loneliness was measured using the University of California, Los Angeles (UCLA) three-item loneliness scale [40]. Geriatric Depression Scale - Short Form (GDS-SF) [41] was used to screen for depression. Participants were also asked to answer the EuroQol-Visual Analogue Scales (EQ-VAS) [42], rating their overall health on a visual analogue scale which spans from 0 (worst) to 100 (best). Risk for malnutrition was assessed using the Mini Nutritional Assessment - Short Form (MNA-SF) [43], which consists of questions pertaining to change in food intake, weight loss, mobility, stress or acute disease, dementia or depression, and BMI. Physical activity was measured as metabolic equivalent of task (MET) per week using the International Physical Activity Questionnaire - Short Form (IPAQ-SF), a self-reported measure of physical activity [44].

### Statistical analysis

As the overall goal of the EASE study was to understand the life space of community-dwelling older adults, sample size was calculated based on the correlation between age and University of Alabama at Birmingham Life Space Activity (UAB-LSA) score in another study [45]. To detect a correlation of 0.1 between age and UAB-LSA score with a power of 0.8 and Type 1 error rate of 0.05, it was determined that at least 900 participants were needed, after accounting for 10% attrition and invalid travel data.

For data analysis, we first performed bivariate linear regressions between each independent variable and BMI as well as Muscle Mass, to understand how each independent variable is associated with the outcome variables by itself (i.e., without covariates). Subsequently, we performed two separate multivariable linear regressions, each with BMI and Muscle Mass as the outcome variable, to examine whether the frequencies of out-of-home activities were significantly associated with BMI and Muscle Mass, controlling for other predictors. In each of these models, we included all the main independent variables of interest (i.e., frequencies of out-of-home dining, grocery shopping, exercise, and recreation) as well as all the covariates (i.e., sociodemographic, environmental, and health variables).

Prior to these analyses, rates of missing data for all variables were reviewed to ensure that they were < 15%, to maintain generalizability of results to the greater population. During the performance of bivariate and multivariable linear regressions, missing datapoints were automatically removed. We excluded extreme outliers above and below three standard deviations from the mean of outcome variables which may unduly influence the linear regression models. This resulted in the removal of 12 and 8 datapoints for the BMI and Muscle Mass models, respectively. The models were validated by conducting a posterior predictive check as well as examining the normality and linearity of residuals, homoscedasticity, and collinearity of independent variables. All analyses were performed using R version 4.3.1.

## Results

Of 1,131 participants enrolled in the project, 13 participants (1.2%) did not complete the 14-day travel survey and were considered to have withdrawn from the study. Analyses were conducted for the remaining 1,118 participants. Only 56 participants (5.0%) opted to record their travels on paper instead of the GPS-based smartphone application. Descriptive statistics for the variables analyzed are presented in Table 1. The mean age of the sample was 63.8 years and 68.0% were female. 91.7% of the sample was of Chinese race, 62.7% had more than secondary education, and 42.9% were retired. For perceived income adequacy, 40.6% said they have enough money with some left over, 43.8% said they had just enough money with no difficulties, 10.2% said they had some difficulty meeting expenses, and 2.3% said they had much difficulty meeting expenses. The proportion of participants with low gait speed was 4.6% and the mean EQ-VAS rating was 83.9. The average total stop counts over 14-days were 8.9 stops for dining out, 5.6 stops for grocery shopping, 4.9 stops for exercise, and 4.0 stops for recreation. The mean BMI was 23.7 kg/m^2^ and mean Muscle Mass was 7.0 kg/m^2^. On average, therefore, the participants had good health and physical function.

**Table 1:** Descriptive statistics (*n* = 1,118)

Variable	Value	Missing, *n* (%)
Total Stop Counts		
Mean Total Stop Count for Dining (SD)	8.9 (6.9)	0 (0)
Mean Total Stop Count for Grocery Shopping (SD)	5.6 (5.0)	0 (0)
Mean Total Stop Count for Exercise (SD)	4.9 (6.1)	0 (0)
Mean Total Stop Count for Recreation (SD)	4.0 (5.6)	0 (0)
Sociodemographic		
Mean Age (SD), *year*	63.8 (7.6)	0 (0)
Gender	-	0 (0)
Female, *n (%)*	760 (68.0)	-
Race	-	0 (0)
Non-Chinese, *n (%)*	93 (8.3)	-
Education	-	0 (0)
More than Secondary Education, *n (%)*	701 (62.7)	-
Employment Status	-	0 (0)
Full-time, *n (%)*	257 (23.0)	-
Part-time, *n (%)*	221 (19.8)	-
Currently not Working, *n (%)*	160 (14.3)	-
Retired, *n (%)*	480 (42.9)	-
Perceived Income Adequacy	-	34 (3.0)
Much difficulty to meet expenses, *n (%)*	26 (2.3)	-
Some difficulty to meet expenses, *n (%)*	114 (10.2)	-
Just enough money, no difficulty, *n (%)*	490 (43.8)	-
Enough money, with some left over, *n (%)*	454 (40.6)	
Housing Type	-	1 (0.1)
Public Housing (1- or 2-room), *n (%)*	95 (8.5)	-
Public Housing (3-room), *n (%)*	118 (10.6)	-
Public Housing (4-room), *n (%)*	283 (25.3)	-
Public Housing (5-room or Executive), *n (%)*	346 (31.0)	-
Condominium/Other Apartments, *n (%)*	169 (15.1)	-
Landed Properties, *n (%)*	104 (9.3)	-
Others, *n (%)*	2 (0.2)	
Marital Status	-	0 (0)
Married, *n (%)*	759 (67.9)	-
Widowed, *n (%)*	80 (7.2)	-
Separated from Spouse, *n (%)*	3 (0.3)	-
Divorced, *n (%)*	83 (7.4)	-
Never Married, *n (%)*	193 (17.3)	-
Mean Number of Household Members (SD)	3.0 (1.4)	0 (0)
Mean LSNS-6 Score (SD)	15.5 (5.5)	129 (11.5)
Environmental		
Mean Distance to Nearest Dining Cluster (SD), *m*	118.4 (91.9)	0 (0)
Mean Distance to Nearest Supermarket (SD), *m*	308.8 (220.1)	0 (0)
Mean Distance to Nearest Wet Market (SD), *m*	1802.4 (1467.2)	0 (0)
Mean Number of Bus Stops in 400 m Radius (SD), *m*	9.6 (3.7)	0 (0)
Mean Distance to Nearest Senior Activity Center (SD), *m*	712.5 (603.0)	0 (0)
Mean Distance to Nearest Park (SD), *m*	168.2 (115.2)	0 (0)
Mean Percentage of Park Areas in 1 km Radius (SD), *%*	7.2 (6.9)	0 (0)
Mean Distance to Nearest Fitness Corner (SD), *m*	319.0 (226.0)	0 (0)
Mean Total Road Network Length in 400 m Radius (SD), *m*	3748.0 (1531.2)	0 (0)
Mean Number of Dwelling Units in 1 km Radius (SD)	22291.0 (7948.3)	0 (0)
Mean NEWS-A Barriers Subscale (SD)	1.7 (0.6)	10 (0.9)
Health		
Gait Speed	-	3 (0.3)
Low, *n (%)*	51 (4.6)	-
Mean Loneliness Score (SD)	2.5 (2.2)	31 (2.8)
Mean GDS-SF Score (SD)	1.9 (2.3)	0 (0)
Mean EQ-VAS Rating (SD)	83.9 (12.1)	0 (0)
Mean MNA-SF Score (SD)	12.6 (1.4)	0 (0)
Mean IPAQ-SF Score (SD), *MET-min/week*	2371.0 (2150.7)	30 (2.7)
Outcome		
Mean BMI (SD), *kg/m*^*2*^	23.7 (4.1)	0 (0)
Underweight (< 18.5 kg/m^2^), *n (%)* Normal weight (18.5–22.9 kg/m^2^) *n (%)* Overweight (23.0–29.9 kg/m^2^) *n (%)* Obese (≥ 30.0 kg/m^2^) *n (%)* Mean Muscle Mass (SD), *kg/m*^*2*^	67 (6.0) 479 (42.8) 488 (43.6) 84 (7.5) 7.0 (1.0)	- - - - 135 (12.1)

*SD *Standard Deviation, *LSNS-6 *6-item Lubben Social Network Scale, *NEWS-A *Neighborhood Environment Walkability Scale-Abbreviated, *GDS-SF *Geriatric Depression Scale - Short Form, *EQ-VAS *EuroQol-Visual Analogue Scales, *MNA-SF *Mini Nutritional Assessment - Short Form, *IPAQ-SF *International Physical Activity Questionnaire - Short Form, *BMI *Body Mass Index

Table 2 shows the results of bivariate linear regression analyses. Most notably in the bivariate analyses, none of the activity stop counts were significantly related to BMI, while dining out, exercise, and recreation stops were significantly related to Muscle Mass. The results of multivariable linear regression analyses for BMI and Muscle Mass are presented in Table 3 and discussed below.

**Table 2:** Bivariate Linear Regression Result Between Each Independent Variable and BMI & Muscle Mass

**Variable**				**BMI**		**Muscle Mass**	
				B*	**P Value**	B*	**P Value**
Total Stop Counts							
	Total Stop Count for Dining			0.02	0.28	0.02	**<0.001**
	Total Stop Count for Grocery Shopping			−0.02	0.29	0.01	0.26
	Total Stop Count for Exercise			−0.02	0.23	0.01	**0.017**
	Total Stop Count for Recreation			0.00	0.84	0.01	**0.022**
Sociodemographic							
	Gender		Female	−0.98	**<0.001**	−1.43	**<0.001**
	Race		Non-Chinese	2.91	**<0.001**	0.28	**0.014**
	Age, *year*			−0.18	0.21	−0.19	**<0.001**
	Education		More than Secondary Education	−0.19	0.41	0.40	**<0.001**
	Employment Status		Part-time	−0.87	**0.011**	−0.40	**<0.001**
			Currently not working	−0.87	**0.021**	−0.49	**<0.001**
			Retired	−0.67	**0.020**	−0.36	**<0.001**
	Perceived Income Adequacy		Some difficulty to meet expenses	−1.05	0.186	0.63	**0.008**
			Just enough money, no difficulty	−2.42	**0.001**	0.26	0.23
			Enough money, with some left over	−2.74	**<0.001**	0.19	0.37
	Housing Type		Public Housing (1- or 2-room)	−1.46	**0.005**	−0.16	0.29
			Public Housing (3-room)	−0.97	**0.030**	0.06	0.66
			Public Housing (4-room)	−1.36	**0.002**	0.02	0.87
			Condominium/Other Apartments	−1.29	**0.008**	0.36	**0.007**
			Landed Properties	−2.19	**<0.001**	−0.04	0.79
			Others	3.51	0.181	0.89	0.20
	Marital Status		Widowed	−0.01	0.97	−0.56	**<0.001**
			Separated from Spouse	2.61	0.22	0.70	0.30
			Divorced	0.03	0.94	−0.37	**0.002**
			Never Married	−0.41	0.174	−0.30	**<0.001**
	Number of Household Members			0.13	0.104	0.09	**<0.001**
	LSNS-6 Score			0.00	0.93	0.00	0.75
Environmental							
	Distance to Nearest Dining Cluster, *500m*			−0.41	0.50	0.56	**<0.001**
	Distance to Nearest Supermarket, *500m*			−0.27	0.29	0.14	**0.045**
	Distance to Nearest Wet Market, *500m*			0.07	0.087	−0.01	0.61
	Number of Bus Stops in 400 m Radius			0.07	**0.019**	−0.01	0.31
	Distance to Nearest Senior Activity Center, *500m*			−0.32	**<0.001**	0.03	0.30
	Distance to Nearest Park, *500m*			−0.42	0.38	0.11	0.44
	Percentage of Park Areas in 1 km Radius, *%*			0.00	0.77	0.00	0.56
	Distance to Nearest Fitness Corner, *500m*			−0.44	0.075	0.11	0.107
	Total Road Network Length in 400 m Radius, *500m*			−0.11	**0.003**	0.00	0.75
	Number of Dwelling Units in 1 km Radius, *1000 units*			0.05	**<0.001**	0.00	0.40
	NEWS-A Barriers Subscale			0.32	0.083	0.10	0.057
Health							
	Gait Speed		Low	2.19	**<0.001**	−0.40	**0.015**
	Loneliness Score			−0.10	**0.045**	−0.01	0.62
	GDS-SF Score			0.16	**<0.001**	0.00	0.93
	EQ-VAS Rating			−0.02	**0.007**	0.00	0.42
	MNA-SF Score			-	-	0.20	**<0.001**
	Muscle Mass, *kg/m*^*2*^			1.65	**<0.001**	-	-
	IPAQ-SF Score, *500 MET-min/week*			−0.07	**0.006**	0.01	0.43

*SD *Standard Deviation, *LSNS-6 *6-item Lubben Social Network Scale, *NEWS-A *Neighborhood Environment Walkability Scale-Abbreviated, *GDS-SF *Geriatric Depression Scale - Short Form, *EQ-VAS *EuroQol-Visual Analogue Scales, *MNA-SF* Mini Nutritional Assessment - Short Form, *IPAQ-SF *International Physical Activity Questionnaire - Short Form, *BMI *Body Mass Index

* B = Unstandardized coefficient

**Table 3:**
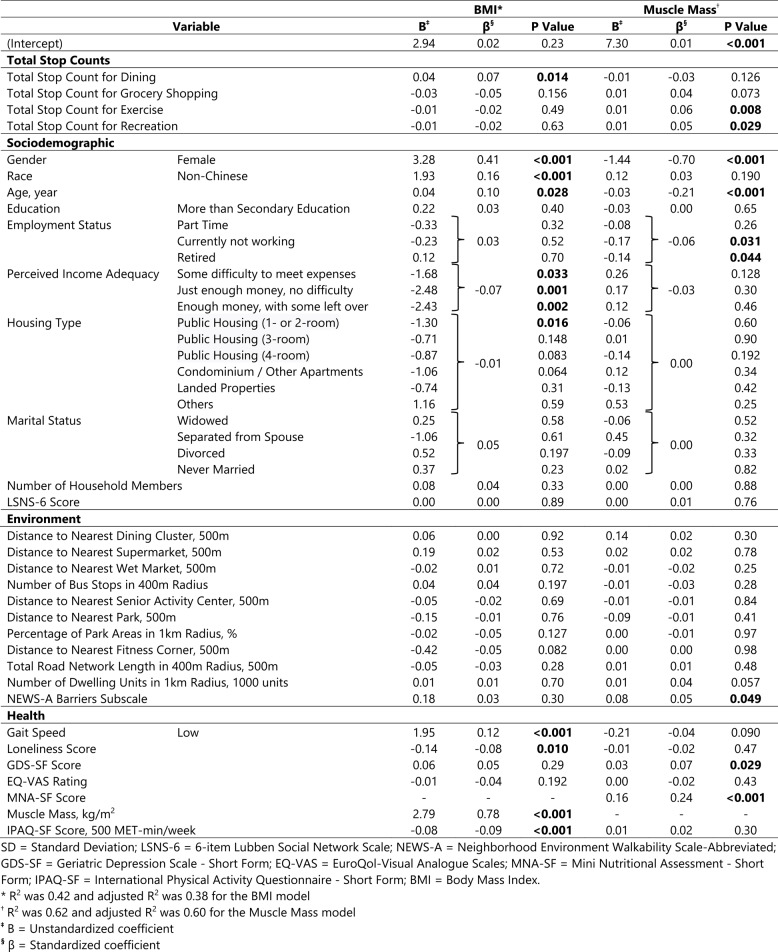
Multivariable Linear Regression Result Between All Independent Variables and BMI & Muscle Mass

### BMI

The R^2^ of the multivariable linear regression model for BMI was 0.42 (adjusted R^2^ = 0.38). With regards to our key activity variables, higher BMI was positively and significantly (*p* < 0.05) associated with increased out-of-home dining frequency, controlling for covariates. Comparing the magnitude of this association with that for age, BMI gained in aging by one year (0.04 kg/m^2^) is approximately equivalent to an increase in one dining stop count over two weeks. A similar relationship pertained to standardized beta coefficients. Other noteworthy statistically significant (*p* < 0.05) predictors include the relationship of better perceived income adequacy to lower BMI. Greater loneliness was significantly associated with lower BMI, while non-Chinese race, female gender, and low gait speed were significantly associated with higher BMI.

### Muscle mass

The R^2^ of the multivariable linear regression model for Muscle Mass was 0.62 (adjusted R^2^ = 0.60). With respect to the activities of our interest, higher Muscle Mass was positively and significantly (*p* < 0.05) associated with more frequent trips for exercise and recreation, controlling for covariates. There was also a strong positive association between grocery stops and Muscle Mass, though not statistically significant. Regarding the effect of activity frequency, approximately three exercise or recreation stops over two weeks is equivalent to the loss of Muscle Mass associated with an increase in age by one year (0.03 kg/m^2^). This relationship held true for standardized beta coefficients as well.

Other noteworthy, statistically significant (*p* < 0.05) predictors included employment status, GDS score, and barriers in neighborhood walkability. Those who were currently not working or retired had lower Muscle Mass compared to those who were working full-time. Surprisingly, a higher GDS score was also associated with higher Muscle Mass. Living in a neighborhood with more hills and/or barriers to walking from place to place was associated with higher Muscle Mass.

## Discussion

We found that frequency of out-of-home daily life activities is associated with clinically relevant differences in BMI and Muscle Mass among community-dwelling older adults. Encouraging older adults to eat home-cooked meals rather than dining out could promote healthier dietary habits and decrease BMI. Furthermore, going outside of the house for recreational and leisure activates, even if not for exercise, may lead to improved Muscle Mass. To the best of our knowledge, this is the first study to explore key daily life activities based on objective GPS-based travel data and their relationship to BMI and Muscle Mass. Key findings for BMI and Muscle Mass are discussed separately.

### BMI

We found that increased out-of-home dining frequency was associated with higher BMI, even after controlling for other predictors including exercise frequency and self-reported measure of physical activity based on IPAQ. This finding may be explained by the known association between out-of-home dining and higher overall energy intake compared to home-cooked meals [46]. However, studies on the relationship between out-of-home dining frequency and BMI show mixed results; while some found a positive association [8, 47], others did not [48, 49]. Our finding provides additional insight as most of the existing studies relied on recall-based food habits, as well as self-reported weight and height and did not consider other relevant life activities and environmental factors.

Regarding the environmental variables, none were significantly related to BMI in the multivariable model. Even in the bivariate model, neither the distance to the nearest dining cluster nor supermarket was significantly associated with BMI. This result is in-keeping with a review article which largely found no relationship between the availability and distance to food outlets and BMI in adults [50].

Other noteworthy findings for BMI include the relationship of low gait speed to higher BMI. While another study also showed a positive association between walking impairments and BMI [23], the causal relationship is likely complex and multifactorial. For example, increased physical activity is associated with higher gait speed [51] and greater weight with increased risks of knee arthritis [52] which can result in lower gait speed. We also found that lower loneliness score was associated with lower BMI. It provides a valuable insight as the association between loneliness and obesity has been inconclusive [23].

Of note, BMI has a well-known “U-shaped” association with adverse health outcomes with increased mortality at the high and low ends, including in Asian populations [53]. Since our focus was on understanding the predictors of BMI, rather than its associated health outcomes, we prioritized the utility in understanding the predictors of incremental differences in BMI rather than broad categories. We note that only 6.0% of participants in our study sample were underweight (< 18.5 kg/m^2^). Nevertheless, we conducted a sensitivity analysis by performing a multivariable regression excluding the underweight participants. The resulting model yielded very similar results to the original model, further supporting the clinical relevance of our findings [see Additional File 1].

### Muscle mass

We found a significant association between increased frequency of out-of-home exercise as well as recreation and higher Muscle Mass. The positive association between out-of-home recreation frequency and Muscle Mass is particularly noteworthy, as it was independent of exercise frequency and self-reported physical activity level based on IPAQ. While physical activity stimulates muscle growth and helps to retain Muscle Mass that declines with age [12], we found that even recreational activities that were not physically vigorous were significantly associated with higher Muscle Mass. Therefore, participating in any recreational activity may be beneficial in preserving Muscle Mass.

Though not statistically significant, greater grocery shopping frequency also showed a strong association with higher Muscle Mass. This could be explained by the fact that dietary protein intake and physical activity are the two most important factors for maintaining Muscle Mass [6]. Frequent grocery shopping may be due to consumption of foods with more protein. Differences in dietary intake between dining out and home cooking warrants further study, particularly in the Singapore context.

For environmental variables, lower neighborhood walkability due to more hills and/or barriers to walking from place to place was significantly associated with higher Muscle Mass. This may be due to an increase in physical activity demand, as suggested in a study examining land slope and physical activity level [54]. Our results showed that other environmental attributes contribute little to Muscle Mass, in keeping with our findings for BMI.

Finally, the positive association between GDS score and Muscle Mass was unexpected as depression is associated with lower Muscle Mass [37]. However, it is unlikely that this association is clinically significant given that the mean GDS score was low.

### Other notable findings

This study was not designed to examine the relationships between various independent variables. Nevertheless, it is intriguing to consider the variables significant in bivariate analyses that became no longer significant in the multivariable analyses. While our bivariate regression showed a significant association between housing type and BMI, the relationship was greatly attenuated once perceived income adequacy is included in the multivariable regression model. This finding is especially interesting in relation to a previous study conducted in Singapore which found that housing type, believed to be a proxy for income, was associated with food insecurity and BMI [55]. In fact, other studies have found a significant association between total household income and BMI [22, 24]. In this study, however, we found that better perceived income adequacy was strongly associated with lower BMI, even after controlling for income surrogates such as housing type, education, and employment status. Therefore, perceived income adequacy may be the important factor, rather than housing itself.

There are two main limitations in our study. One is that we are unable to infer the directionality of association between the dependent and independent variables due to the cross-sectional study design. For example, while it is reasonable to hypothesize that those who go out more for exercise and recreation have a higher Muscle Mass because of increased physical activity, one could also argue that a higher Muscle Mass enables increased participation in exercise and recreation; the reality is probably a mixture of both. The other limitation of our study is the lack of diet history, including the types of food outlets participants visited and the food they consumed. As a result, we cannot delineate the dietary and nutritional mechanisms by which dining out and grocery shopping frequency may relate to BMI and Muscle Mass. Despite these limitations, however, the large sample size and comprehensive range of variables enhance the generalizability of our findings to the broader population of community-dwelling older adults in urban settings.

## Conclusion

This study identified key life activities associated with clinically significant differences in BMI and Muscle Mass among community-dwelling older adults, including the frequencies of out-of-home dining, exercise, and recreational activities. We found that participation in these specific life activities, controlling for sociodemographic, health, and environmental factors, was differentially associated with BMI and Muscle Mass.

## Supplementary Information

Supplementary Material 1

## Data Availability

The data generated during and/or analyzed during the current study are not currently publicly available.

## Abbreviations

BIA: Bioelectrical impedance analysis
BMI: Body Mass Index
DBSCAN: Density-Based Spatial Clustering of Applications with Noise
EASE: Elderly Life Activity-Space
EQ-VAS: EuroQol-Visual Analogue Scales
GDS: Geriatric Depression Scale
GPS: Global Positioning System
IPAQ: International Physical Activity Questionnaire
LSNS-6: 6-item Lubben Social Network Scale
MET: Metabolic Equivalent of Task
IPAQ: International Physical Activity Questionnaire
MNA: Mini Nutritional Assessment
NEWS-A: Neighborhood Environment Walkability Scale-Abbreviated
SD: Standard Deviation
SF: Short Form
UAB-LSA: University of Alabama at Birmingham Life Space Activity
UCLA: University of California, Los Angeles
