# Supplementary material for: Out-of-home activity participation associated with body mass index and muscle mass in community-dwelling older adults
Source: BMC Public Health. 2025 Dec 12;26:115. doi: 10.1186/s12889-025-24813-7 (PMC12781809; doi:10.1186/s12889-025-24813-7)
Supplement: Supplementary file 1 — Supplementary Material 1 [file 12889_2025_24813_MOESM1_ESM.docx]

**Additional File 1**

**Table 4. Multivariable Linear Regression Result Between All Independent Variables and BMI [Excluding Underweight Participants]**

|  | | **BMI*** | | |
| --- | --- | --- | --- | --- |
| **Variable** | | **B^‡^** | **β^§^** | **P Value** |
| (Intercept) | | 6.01 | 0.01 | 0.016 |
| Total Stop Count for Dining | | 0.03 | 0.06 | **0.040** |
| Total Stop Count for Grocery Shopping | | -0.04 | -0.06 | 0.085 |
| Total Stop Count for Exercise | | -0.01 | -0.02 | 0.46 |
| Total Stop Count for Recreation | | 0.00 | 0.00 | 0.96 |
| Gender | Female | 2.98 | 0.40 | **<0.001** |
| Race | Non-Chinese | 1.71 | 0.15 | **<0.001** |
| Age, *year* |  | 0.03 | 0.08 | 0.122 |
| Education | More than Secondary Education | 0.25 | 0.04 | 0.35 |
| Employment Status | Part Time | -0.24 | 0.03 | 0.47 |
|  | Currently not working | -0.19 |  | 0.60 |
|  | Retired | 0.13 |  | 0.68 |
| Perceived Income Adequacy | Some difficulty to meet expenses | -1.35 | -0.08 | 0.079 |
|  | Just enough money, no difficulty | -2.04 |  | **0.006** |
|  | Enough money, with some left over | -2.05 |  | **0.006** |
| Housing Type | Public Housing (1- or 2-room) | -1.26 | -0.04 | **0.022** |
|  | Public Housing (3-room) | -1.02 |  | **0.039** |
|  | Public Housing (4-room) | -1.08 |  | **0.031** |
|  | Condominium / Other Apartments | -1.35 |  | **0.018** |
|  | Landed Properties | -0.93 |  | 0.196 |
|  | Others | 0.85 |  | 0.68 |
| Marital Status | Widowed | 0.31 | 0.05 | 0.51 |
|  | Separated from Spouse | -0.96 |  | 0.64 |
|  | Divorced | 0.51 |  | 0.199 |
|  | Never Married | 0.42 |  | 0.179 |
| Number of Household Members | | 0.07 | 0.03 | 0.40 |
| LSNS-6 Score | | -0.01 | -0.01 | 0.67 |
| **Environment** | |  |  |  |
| Distance to Nearest Dining Cluster, *500m* | | -0.25 | -0.02 | 0.69 |
| Distance to Nearest Supermarket, *500m* | | 0.34 | 0.04 | 0.30 |
| Distance to Nearest Wet Market, *500m* | | -0.04 | 0.00 | 0.44 |
| Number of Bus Stops in 400m Radius | | 0.04 | 0.04 | 0.20 |
| Distance to Nearest Senior Activity Center, *500m* | | 0.00 | -0.01 | 0.97 |
| Distance to Nearest Park, *500m* | | -0.36 | -0.03 | 0.45 |
| Percentage of Park Areas in 1km Radius, *%* | | -0.03 | -0.06 | 0.062 |
| Distance to Nearest Fitness Corner, *500m* | | -0.44 | -0.06 | 0.070 |
| Total Road Network Length in 400m Radius, *500m* | | -0.05 | -0.03 | 0.29 |
| Number of Dwelling Units in 1km Radius, *1000 units* | | 0.01 | 0.02 | 0.56 |
| NEWS-A Barriers Subscale | | 0.20 | 0.03 | 0.24 |
| Gait Speed | Low | 2.09 | 0.13 | **<0.001** |
| Loneliness Score | | -0.12 | -0.07 | **0.031** |
| GDS-SF Score | | 0.06 | 0.05 | 0.31 |
| EQ-VAS Rating | | -0.01 | -0.04 | 0.193 |
| MNA-SF Score | | - | - | - |
| Muscle Mass, *kg/m^2^* | | 2.51 | 0.74 | **<0.001** |
| IPAQ-SF Score, *500 MET-min/week* | | -0.06 | -0.08 | **0.007** |

SD = Standard Deviation; LSNS-6 = 6-item Lubben Social Network Scale; NEWS-A = Neighborhood Environment Walkability Scale-Abbreviated; GDS-SF = Geriatric Depression Scale - Short Form; EQ-VAS = EuroQol-Visual Analogue Scales; MNA-SF = Mini Nutritional Assessment - Short Form; IPAQ-SF = International Physical Activity Questionnaire - Short Form; BMI = Body Mass Index.

* R^2^ was 0.38 and adjusted R^2^ was 0.35.

**^‡^** B = Unstandardized coefficient.

**^§^** β = Standardized coefficient.
